# Coronectomy of mandibular third molars with dental pathology: a prospective cohort study of 121 molars

**DOI:** 10.1007/s10006-025-01340-8

**Published:** 2025-01-24

**Authors:** Arjan van Bodegraven, Rashida N. Simons, Jacco G. Tuk, Jan de Lange, Jerome A. H. Lindeboom

**Affiliations:** 1 Department of Oral and Maxillofacial Surgery, Amsterdam UMC, University of Amsterdam, Amsterdam, The Netherlands; 2 Private Practice, Amstelland Hospital, Amstelveen, The Netherlands

**Keywords:** Coronectomy, Cyst, Pathology, Caries, Mandibular third molar

## Abstract

**Purpose:**

Coronectomy is a valuable treatment proven safe for non-pathological mandibular third molars with an increased risk of inferior alveolar nerve injury. Coronectomy may also be useful for mandibular third molars with dentigerous cysts and caries, but this is not commonly performed due to the lack of well-designed, evidence-based studies. Here, we aim to investigate the safety of coronectomy for mandibular third molars with caries and dentigerous cysts.

**Methods:**

One hundred fifteen patients with an impacted mandibular third molar with a dentigerous cyst or caries underwent coronectomy or complete removal and received follow-up with clinical and radiographical examinations. We statistically compared the presence of postoperative complications after coronectomy versus complete removal.

**Results:**

Data from 121 molars were available for analysis. The results revealed no significant difference in the occurrence of postoperative complications (including persistent pain, inferior alveolar nerve injury, infection, alveolar osteitis, excessive bleeding, and the need for retreatment) between coronectomy and complete removal. Additionally, the incidence of postoperative complications was not related to any analyzed patient- or molar-related factors, including age, gender, health status, smoking, caries, dentigerous cyst extent, and impaction degree.

**Conclusion:**

For pathological impacted mandibular third molars with dentigerous cysts and caries, coronectomy exhibited both short-term and long-term safety, showing no significant difference in postoperative complications compared to total removal. Our results suggest that coronectomy can be indicated for pathological mandibular third molars with proximity to the inferior alveolar nerve.

**Trial registration number:**

Not applicable.

## Introduction

Mandibular third molar (MTM) removal is a standard surgical procedure in dentistry. However, when the MTMs are in proximity to the inferior alveolar nerve (IAN), this procedure carries an increased risk of IAN injury (IANI) [1]. This risk can be reduced by performing a coronectomy on the MTM [2, 3]. Coronectomy involves removing the crown of the MTM while the roots stay intact in the alveolar bone [4]. Increasing evidence supports the safety of the coronectomy procedure on non-pathological MTMs [5], and there remains a need to assess the safety of additional applications of coronectomy. For example, it is still unclear whether coronectomy can be safely performed in patients with MTMs with pathologies, such as dentigerous cysts or caries.

Dentigerous cysts are odontogenic cysts attached to the cementoenamel junction of an unerupted tooth crown. They result from alteration of the reduced enamel epithelium after completion of amelogenesis, which leads to fluid accumulation between the epithelium and tooth crown [6–8]. Radiographically, dentigerous cysts typically present with well-defined unilocular radiolucency of at least 3–4 mm diameter around the crown of an unerupted tooth [6–8]. Imaging is insufficient for dentigerous cyst diagnosis [8], and histopathological examination is required. Infected dentigerous cysts can cause pain and swelling. Moreover, expanded dentigerous cysts may affect the prognosis of adjacent molars [6–8] and, in rare cases, predispose the patient to a pathological jaw fracture [7].

Dental caries is a localized chemical dissolution of the tooth surface caused by metabolic events in the biofilm (dental plaque) covering the affected area. The consequent destruction of enamel, dentin, and cementum results in tooth decay [9]. Deep caries is an advanced carious lesion, where exposure to dental pulp and pulp inflammation is possible, based on clinical and radiographic information [9, 10].

A successful coronectomy requires a vital root without pulpal inflammation and healthy alveolar bone [11]. Hence, coronectomy is theoretically contraindicated (not evidence-based) in teeth with probable pulpal infection due to caries or a dentigerous cyst; thus, such teeth are completely removed [11–13].

Patel et al. [14] and Henien et al. [15] reviewed instances of coronectomy on teeth with dentigerous cysts in a case series of 21 and 68 patients, respectively. Patel et al. [13] also reviewed a case series of 14 carious teeth that underwent coronectomy. These reviews demonstrated the successful use of coronectomy to treat carious teeth and teeth with dentigerous cysts [13–15]. However, despite these findings, there is insufficient evidence to prove the short-term and long-term safety of coronectomy in teeth with dental pathologies.

In the present study, we aimed to investigate the safety of coronectomy of impacted MTMs with dental caries and dentigerous cysts. We hypothesized that coronectomy could be safely performed on MTMs with pathology. This is the first prospective cohort study examining coronectomy for pathological MTMs and the most extensive study on this subject.

## Materials and methods

### Ethical considerations

This study was approved by the Medical Ethical Committee of Amsterdam University Medical Center (reference number 266#21.292) and the ACTA Ethics Committee (reference number 2023–49571). Every patient was given detailed information about the study, and all patients gave signed consent to be included in the study and agreed to appear for the surgery and follow-up session(s).

### Eligible patients

All patients included in this study were referred by their dental general practitioner to the Oral and Maxillofacial department of the Amstelland Hospital in Amstelveen, The Netherlands, for MTM management between March 2015 and September 2023. This study included patients of all ages and genders who were scheduled for surgical treatment of an impacted MTM with an increased risk of IANI and who had a (histopathological) diagnosis of a dentigerous cyst or caries. In patients with a presumed diagnosis of a dentigerous cyst, an incisional biopsy was performed to have a definite histopathological diagnosis of a dentigerous cyst and to exclude other pathologies such as keratocyst or ameloblastoma. Exclusion criteria were as follows: age of < 18 years; failure to show up for postoperative follow-up; history of kidney failure, hematological disorder, or any form of chronic hepatic disease; history of gastric ulcers; diagnosis with uncontrolled diabetes; current pregnancy or lactation; infection with human immunodeficiency virus (HIV); prior radiation therapy to the maxillofacial region; prior organ or marrow transplantation (donors and recipients); and corticosteroid use within 15 days before surgery.

### Radiographical assessment

Every patient underwent preoperative radiographic orthopantomography (X-OPT) assessment with the Orthopantomograph ^®^ OP100 D (GE Healthcare, Dental, Tuusula, Finland) used for all recordings before January 22, 2021, and the Sirona Orthophos SL 2D (Dentsply Sirona, Charlotte, USA) used for recordings after January 22, 2021. X-OPT imaging was essential for analyzing the grade of MTM impaction, determined based on Pell & Gregory’s classification [16], the proximity of the MTM to the inferior alveolar canal, and the extent of MTM pathology.

When X-OPT revealed the proximity of the MTM to the inferior alveolar canal, the following seven radiographic signs were used to evaluate the likelihood of IANI [17, 18]:

- Darkening of the root.
- Deflected roots.
- Narrowing of the root.
- Dark and bifid root.
- Interruption of the white opaque line(s) representing the inferior alveolar canal.
- Diversion of the inferior alveolar canal.
- Narrowing of the inferior alveolar canal.

Patients having one or more of these radiographical indicators were referred for additional computed tomography (X-CT) (Philips Ingenuity 128 CT Scanner, Integrity Medical, Fort Myers, Florida, USA) to visualize the relationship of the MTM to the inferior alveolar canal in all three dimensions [19–21]. The DICOM X-OPT and X-CT files were viewed using Phillips Healthcare software for Picture Archiving and Communication System (PACS, Phillips Healthcare, Phillips IntelliSpace PACS Enterprise 4.4).

Total removal carried a higher likelihood of IANI when X-CT confirmed one of the following three anatomical situations [22–25]:

- Lingual position of the inferior alveolar canal relative to the radices of the MTM with flattening of the inferior alveolar canal;
- Flattening of the inferior alveolar canal at the contact surface with the MTM’s radices; and.
- Inter-radicular position of the inferior alveolar canal surrounded by the MTM’s radices.

In this study, when X-CT confirmed one of these situations, coronectomy was performed as an alternative to total removal to prevent IANI [2, 3]. Subjects without increased risk of IANI based on X-CT imaging were treated with complete removal. The radiographical findings and treatment planning were explained to the patients, and treatment was initiated with the patient’s consent.

**Fig. 1:**
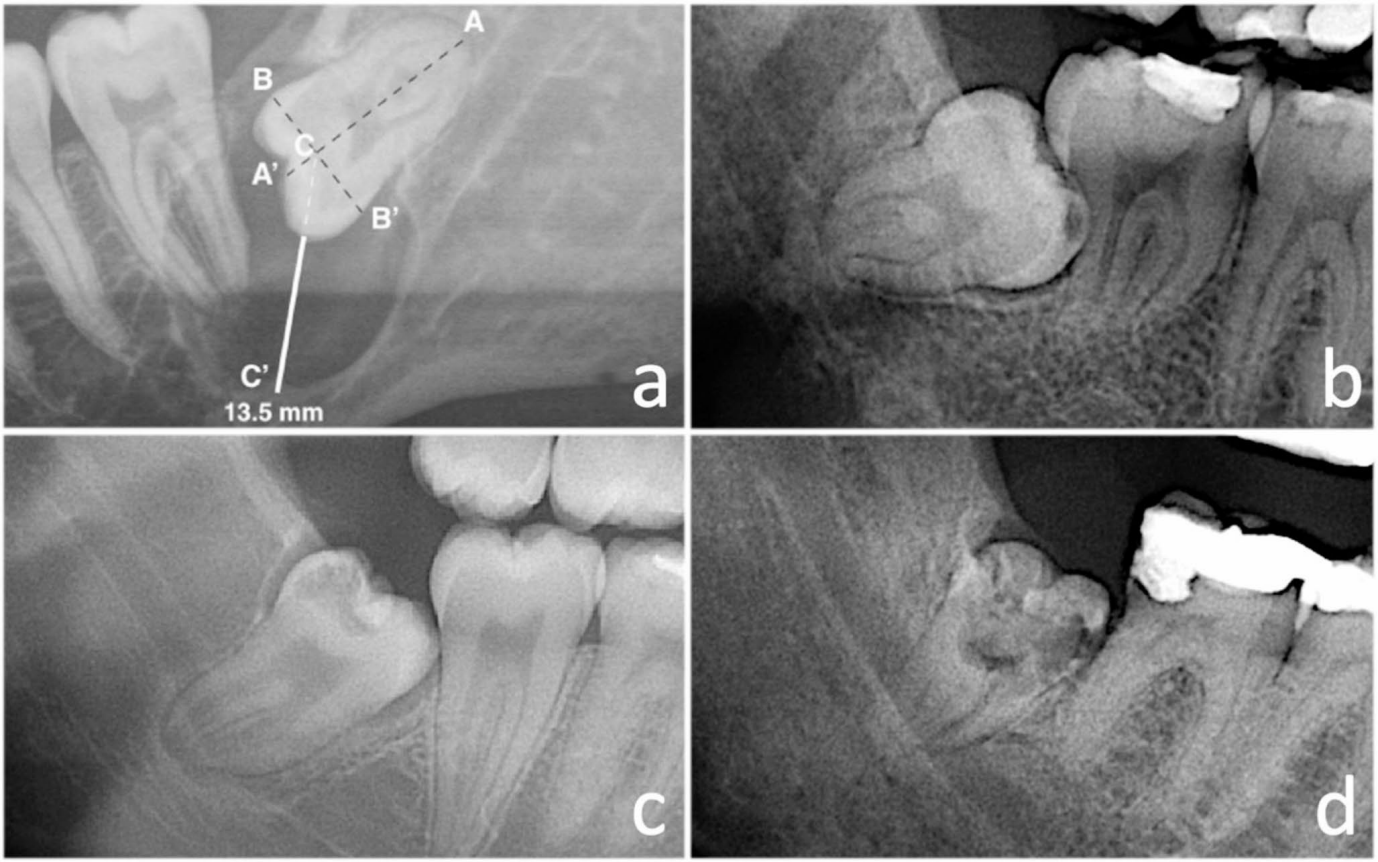
**a**. Method to determine the size of the pericoronal radiolucent area (C-C’). **b**-**d**. Grades of caries lesions: grade **A**, carious lesion’s radiolucency restricted to enamel (**b**); grade **B**, caries’ radiolucency extending into dentin (**c**); and grade C, caries’ radiolucency extending into the pulp (**d**)

X-OPT was also used to investigate the pathology of MTMs. In cases where dentigerous cysts were suspected on the MTMs, X-OPT revealed a well-defined unilocular crown-related radiolucency. The radiolucency size was estimated using the ruler measuring tool of the PACS, following the method described by Damante & Fleury [26]. Two perpendicular lines (A-A’ and B-B’) were drawn on the molar; one across the molar’s long axis and the other mesiodistally across the crown. Line C-C’ was drawn through the junction of A-A’ and B-B’ to the widest point of the radiolucency. The distance in millimeters from the crown’s edge to the outermost radiolucency was estimated using the digital ruler in line with C-C’ (Fig. 1a). The MTMs were classified into two groups according to the cyst‘s extensiveness: <1 cm and > 1 cm. X-OPT was also used to diagnose caries and to determine the extent of the lesion in three grades: A, radiolucency restricted to the enamel; B, radiolucency extending into dentin; and C, radiolucency extending into the pulp (Fig. 1b-d).

### Surgical procedure

The surgical procedures were performed using a similar technique by two oral and maxillofacial surgeons with over twenty years of experience in dentoalveolar surgery. Every patient received a mandibular nerve block injection and buccal nerve infiltration. Hospital protocol included standardization of the anesthesia type- and dosage (40 mg articaine/hydrochloride with 0.01 mg epinephrine, administered with a 1.7 mL syringe, Ultracain D-S forte; Sanofi-Aventis, Netherlands BV, Gouda, the Netherlands), type of needle (27 gauge/0.40 × 35 mm), area of administering, temperature, and repository.

Subjects with an increased risk of IANI were treated with coronectomy following the procedure described by Pogrel et al. [4]. In all cases, a triangle flap design was performed. Next, a round surgical bur was used to remove bone overlaying the impacted MTM until the cementoenamel junction was exposed. A steel surgical fissure bur separated the MTM crown from the roots. The roots were reduced to 3–4 mm below the alveolar crest height. Excessive irrigation with a sterile distilled water solution was used when using the surgical handpiece.

Subjects without increased risk of IANI, based on X-CT, were treated with complete surgical MTM removal using the techniques described by Pell & Gregory [16]. In all cases, a triangle flap design was performed. A round steel surgical bur was used to remove all bone overlaying the impacted MTM until the cementoenamel junction was. A surgical steel fissure bur was used to section the molar into pieces to be removed. Excessive irrigation with sterile distilled water was used when using the surgical handpiece.

The cysts were enucleated in patients with dentigerous cysts, and tissue was submitted to the pathology department to confirm the original diagnosis.

The surgical site was primarily sutured with 3/0 Undyed Vicryl Rapide (Ethicon, Somerville, MA, USA). Details about the surgery were reported in the digital patient record immediately after the procedure.

### Postoperative management

Every patient was given strict postoperative instructions to bite on gauze for 30 min and not to rinse their mouth or spit for 24 h. Patients were prescribed ibuprofen (Brufen 600 mg, Abbott BV, Hoofddorp, the Netherlands) as an analgesic to take three times daily starting within two hours after surgery, while in older patients (> 60 years old), 1000 mg paracetamol, four times a day was prescribed. Additionally, 0.12% chlorhexidine mouthwash (Perio•Aid Intensive Care ^®^, Dentaid Benelux BV, Houten, the Netherlands) was used as an antiseptic, for one minute three times daily, for five days, starting 24 h postoperatively. The patients in the surgical removal group were given a Monoject Curved 412 Tip Syringe (Tyco/Healthcare-Kendall, Mansfield, MA) to rinse the surgical site three times daily starting on postoperative day three. Antibiotics were not provided in a standardized fashion but were instead prescribed only to patients with large cysts, deep caries, and those with a related health status. Postoperative instructions were given verbally and in writing to every patient.

### Data collection

After either surgical procedure, patients were assessed for the following postoperative complications:

- Sensibility disorder of the IAN.
- Alveolar osteitis.
- Surgical site infection.
- Persistent pain (pain > 1 week).
- Excessive bleeding.
- The need for retreatment.

Postoperative clinical and radiographical follow-up was assessed by the two oral- and maxillofacial surgeons who performed the surgery. Patients were examined for sensibility disorders of the IAN at two-, six- and twelve months postoperatively. A two-point discrimination and static light touch detection test [27] examined sensory abnormalities such as pain, numbness, paresthesia, or dysesthesia. Alveolar osteitis, postoperative infection, bleeding, and persistent pain for longer than one week were examined and recorded after reviewing the patients’ self-reported problems, which participants had agreed to complete. The need for retreatment after coronectomy was analyzed using X-OPT to determine coronal migration of the roots, and a periodontal probe was used to assess the roots’ impaction by overlying bone [28].

Data were collected and stored in a database. The variables were primarily used for descriptive purposes and secondary for data analysis. The demographic variables were age, computed as the interval in years between the date of surgery and the participant’s date of birth, and gender, noted as the gender recorded at the participant’s birth (male/female). The medical anamnesis was completed on paper, including the variables of smoking and health status (ASA: American Society of Anesthesiologists). The demographic and medical data at the first consultancy were documented in the digital patient record. Anatomic variables included the MTMs impaction degree according to the classification of Pell & Gregory [16]: the cyst size estimated with X-OPT (Fig. 1a): and the extent of caries determined with X-OPT (Fig. 1b-d).

### Statistical analysis

Fisher’s exact test was used to assess the significance of the relationship between treatment (coronectomy or complete removal) and the incidence of postoperative complications (IANI, alveolar osteitis, surgical site infection, persistent pain, and bleeding). These analyses were performed individually for the dentigerous cyst and caries groups. The relationships between the incidence of postoperative complications and patient- and molar-related factors were statistically assessed using Fisher’s exact test for the factors age, gender, smoking, impaction degree, and the cyst size; and with the Mann Whitney U test for the factors ASA score and extent of caries. The use of postoperative antibiotics was statistically assessed using the Mann-Whitney U test for the factors of ASA score and extent of caries, the Chi-square test for the impaction degree, and Fisher’s exact test for the cyst size.

When the Chi-square test’s statistical outcomes had an expected count of less than 5 in more than 20% of the cells, the P-value of the Fisher’s Exact Test was used to assess the significance. Statistical analyses were performed at the MTM level, not the patient level. For patients who underwent treatment for both left and right pathological MTMs, the analyses included both MTMs. All statistical analyses were performed using the software Statistical Package for Social Sciences-SPSS (IBM Inc., USA) version 27.0. A significance level of less than 0.05 was applied to the probability.

## Results

Data were reported from 115 patients, including 66 males and 49 females, of 18–91 years of age (mean: 44.4 years, SD: 18.9 years) (Table 1). A total of 121 MTMs were available for statistical analyses. Based on X-CT findings, 69 MTMs (57%) were assessed to have an increased risk of IANI and, therefore, were treated by coronectomy. The remaining 52 MTMs (43%) were removed entirely. A total of 63 MTMs were suspected to involve a cyst based on the X-OPT, which was preoperatively confirmed with pathological examination. 61 MTMs of the cyst group were unerupted, and two were partially erupted. When these cysts were classified according to their estimated extensiveness, 52 MTMs (83%) had a cyst smaller than 1 cm, and 11 MTMs (18%) had a cyst larger than 1 cm. A total of 58 carious MTMs were treated. Three MTMs (5%) had caries in the enamel layer, 26 (45%) had caries into the dentin, and 29 (50%) had caries extending into the dental pulp. The degree of MTM impaction ranged from grade IA to IIIC, according to the classification of Pell & Gregory [16]. A total of 47 MTMs (39%) were classified as degree A (IA, IB, IIA, IIB) and 74 (61%) as degree B for deep impaction (IC, IIC, IIIA, IIIB, IIIC).

**Table 1:** Patient demographics and mandibular third molar characteristics

Patients; *N*	115
Gender	
Male; N (%)	66 (57)
Female; N (%)	49 (43)
Age (years); mean (Min.-Max.)	44.4 (18–91)
Smoking; N (%)	12 (10)
ASA score	
ASA 1; N (%) ASA 2; N (%) ASA 3; N (%)	82 (71) 26 (23) 7 (6)
**Mandibular third molars; N**	**121**
**MTM with dentigerous cysts; N**	**63**
Size of cyst	
< 1 cm; N (%) > 1 cm; N (%)	52 (83) 11 (17)
Impaction degree*	
A. 1B, 2 A, 2B; N (%) B. 1 C, 2 C, 3 A, 3B, 3 C; N (%)	22 (35) 41 (65)
Treated with coronectomy; N (%)	38 (60)
Treated with total removal; N (%)	25 (40)
**MTM with caries; N**	**58**
Extent of caries**	
A. enamel; N (%) B. dentin; N (%) C. dental pulp; N (%)	3 (5) 26 (45) 29 (50)
Impaction degree*	
A. 1A, 1B, 2 A, 2B; N (%) B. 1C, 2 C, 3 A, 3B, 3 C; N (%)	25 (43) 33 (57)
Treated with coronectomy; N (%)	31 (53)
Treated with total removal; N (%)	27 (47)

*Impaction degree following Pell and Gregory [16]. **Caries extent A, B, and C, as illustrated in Fig. 1b-d

MTM: Mandibular third molar, N: Number, ASA: American Society of Anesthesiologists

Among the patients diagnosed with a dentigerous cyst, one (3%) reported pain lasting longer than one week after coronectomy and two (8%) after total removal. Among the patients with caries, four (13%) experienced persistent pain (pain > 1 week) after coronectomy and one (4%) after complete removal. The pain did not significantly differ between coronectomy versus complete removal among patients with a dentigerous cyst (*p* = 0.557) or those with caries (*p* = 0.359). All patients experiencing persistent pain underwent an examination, revealing no evidence of infection or alveolar osteitis. The wound was irrigated with a sodium chloride solution using a Monoject^®^ syringe, and patients were instructed to rinse the socket three times daily for the next five days. Five patients were prescribed amoxicillin 500 mg, three times daily, for five days, while one patient received doxycycline 200 mg on the first day, followed by 100 mg once daily for seven days. One patient, who was diagnosed with a dentigerous cyst and who underwent complete removal, reported pain three months after surgery. Examination revealed the presence of an erupting bony sequester, which was successfully removed using a minimally invasive technique. Following this intervention, the patient experienced a full recovery, and the pain disappeared.

Two patients (5%), each with a dentigerous cyst, were diagnosed with postoperative hypo-anesthesia of the IAN and experienced numbness of the chin and lower lip after the coronectomy. Both patients recovered within six months. The occurrence of IANI did not significantly differ between coronectomy and total removal (*p* = 0.514). No occurrence of IANI was recorded in the caries group.

Postoperative infection, alveolar osteitis, and bleeding were not observed in the caries group or the dentigerous cyst group after either type of surgery. Among all patients diagnosed with caries, two (7%) retained roots were accessible with a periodontal probe and showed vertical migration on the X-OPT at the six-month follow-up after coronectomy. These indicators suggested that the residual roots were not impacted in the jaw by overlying bone; thus, secondary surgery was required to remove them completely. The two repeated surgeries did not result in any IANI. The six-month follow-up was not attended by four patients in the caries group and three patients in the dentigerous cyst group. These non-responders were excluded from the long-term follow-up analysis.

None of the patient- and molar-related factors were significantly related to the incidence of postoperative complications (Table 2).

**Table 2:** Patient- and molar-related factors related to the incidence of postoperative complications

		MTMS with dentigerous cysts (*N* = 63)			MTMS with caries (*N* = 58)		
		Postoperative complications*		*p*	Postoperative complications*		*p*
		Yes (*N* = 5)	No (*N* = 58)		Yes (*N* = 5)	No (*N* = 53)	
**AGE; N (%)**							
≤ 30 years old		1 (2)	24 (38)	0.640^*ǂ*^	1 (2)	19 (33)	0.650^*ǂ*^
> 30 years old		4 (6)	34 (54)		4 (7)	34 (59)	
**GENDER; N (%)**							
Male		2 (3)	37 (59)	0.360^*ǂ*^	4 (7)	28 (48)	0.*367*^*ǂ*^
Female		3 (5)	21 (33)		1 (2)	25 (43)	
**SMOKING; N (%)**							
Yes		1 (2)	13 (21)	1.000^*ǂ*^	1 (2)	9 (16)	1.000^*ǂ*^
No		4 (6)	45 (71)		4 (7)	44 (76)	
**ASA score; N (%)**							
ASA 1		5 (8)	46 (73)	0.263^*§*^	2 (3)	34 (59)	0.273^*§*^
ASA 2		0 (0)	11 (17)		2 (3)	14 (24)	
ASA 3		0 (0)	1 (2)		1 (2)	5 (9)	
**IMPACTION DEGREE; N (%)**							
A. 1B, 2A, 2B; N (%)		2 (3)	20 (32)	1.000^*ǂ*^	2 (3)	23 (40)	1.000^*ǂ*^
B. 1C, 2C, 3A, 3B, 3 C; N (%)		3 (5)	38 (60)		3 (5)	30 (52)	
**EXTENT OF CARIES; N (%)**							
A. enamel					0 (0)	3 (5)	0.584^*§*^
B. dentin					2 (3)	24 (41)	
C. pulp					3 (5)	26 (45)	
**SIZE CYST; N (%)**							
< 1 cm		4 (6)	48 (76)	1.000^*ǂ*^			
> 1 cm		1 (2)	10 (16)				

Statistical analyses were performed at MTM level, not at the patient level. *The total occurrence of the following postoperative complications: IANI, alveolar osteitis, surgical site infection, persistent pain, and excessive bleeding

^ǂ^Fisher’s exact test, ^§^Mann Whitney U test, MTMs: Mandibular Third Molars, ASA: American Society of Anesthesiologists

**Fig. 2:**
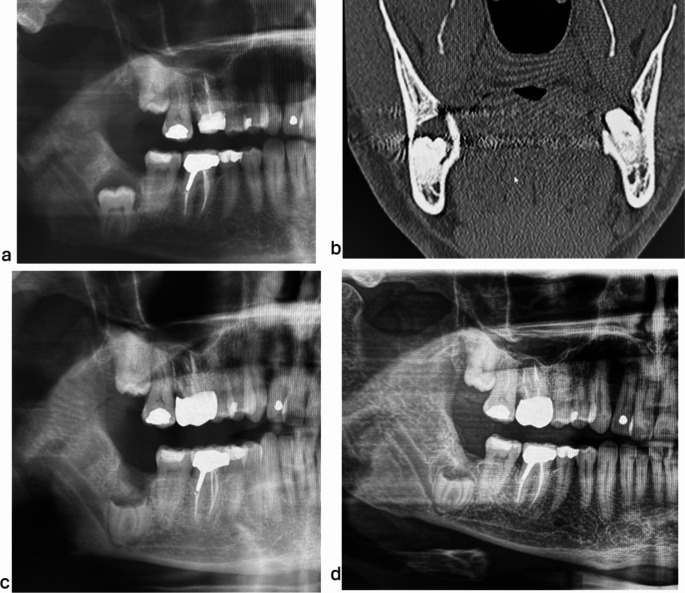
(**a**) Preoperative X-OPT of impacted right mandibular third molar with a coronal radiolucency. (**b**) Preoperative X-CT coronal slice of impacted right mandibular third molar with a coronal radiolucency. (**c**) X-OPT 6 months after coronectomy of the mandibular third molar. (**d**) X-OPT 2 years after coronectomy of the mandibular third molar showing bony healing

In the caries group, we found that the number of patients prescribed antibiotics significantly correlated with the ASA score (Z = -2.390 and *p* = 0.017) and the extent of caries (Z = -2.502 and *p* = 0.012). Antibiotics were more commonly prescribed for patients with a higher ASA score and those with larger cysts. Figure 2 shows the preoperative and postoperative radiographs of a patient with a dentigerous cyst who underwent a coronectomy of an impacted mandibular third molar.

## Discussion

This study aimed to investigate the safety of the coronectomy procedure for impacted MTMs with dental caries and dentigerous cysts. Our results showed that the rate of complications after coronectomy did not significantly differ from the gold standard, which is complete removal.

Dentigerous cysts most often develop at the age of 10–30, which is lower than the mean age of our cohort (42.0 years; range 18–76 years, SD:17.1). This suggests that the patients in this study may have been undiagnosed for a long time, probably due to the cyst’s asymptomatic course [7, 8].

In the present study, patients with a presumed clinical and radiological diagnosis of dentigerous cyst underwent a biopsy to establish a definite diagnosis. A biopsy is crucial for diagnosing a dentigerous cyst because it helps ensure accurate identification of the lesion, guides appropriate treatment, and prevents potential complications. Dentigerous cysts mimic other lesions or pathologies, including more aggressive conditions such as odontogenic keratocysts, ameloblastomas, or even malignant tumors [8]. Relying solely on clinical or radiographic findings may lead to misdiagnosis, resulting in inadequate or excessive treatment.There are two approaches for treating dentigerous cysts: enucleation and marsupialization. In the present study, all the cysts were enucleated. Although marsupialization reduces the risks of complications, The disadvantage of marsupialization is the potential for residual pathological tissue, which in rare cases may involve ameloblastoma or mucoepidermoid carcinoma [29]. The coronectomy makes it possible to completely remove the cyst lining and prevents recurrence by removing its attachment to the tooth at the cement-enamel junction [15].

In a recent study, pain and Oral Health-Related Quality of Life (OHRQoL) were compared between patients after coronectomy and complete surgical removal of mandibular third molars during the first postoperative week. Coronectomy significantly impacted postoperative oral health-related OHRQoL more compared to the complete surgical removal of mandibular third molars [30]. In the present study, only a low number of patients experienced persistent postoperative pain in the dentigerous cyst group; one patient (3%) experienced pain for over one week after coronectomy, and two (8%) after complete removal (*p* = 0.557). A slightly larger (but still low) number of patients experienced persistent postoperative pain in the caries group; four patients (13%) experienced persistent pain after coronectomy, and one (4%) after complete removal (*p* = 0.359). Other studies have reported higher rates of patients with persistent pain after removal of non-pathological MTMs, ranging from 13.8 to 31.2% after coronectomy and 15.2–57.3% after removal [2, 3, 31–33]. Among the eight patients in our study who reported persistent postoperative pain, six were provided antibiotics (five were prescribed amoxicillin 500 mg three times daily for five days; one received doxycycline starting with two times 100 mg followed by once daily for seven days). Examination of these cases revealed no signs of postoperative infection or alveolar osteitis. It can be questioned whether antibiotics are needed for patients reporting persistent pain. In theory, patients with persistent pain may be at risk of impaired wound healing, possibly due to the presence of bacteria. Antibiotics could enhance wound healing by lowering the number of bacteria in the wound area, thereby depressing postoperative infection [34]. However, the impact of antibiotics on treating persistent pain remains controversial.

The bleeding rates in this study were minimal since no cases of postoperative bleeding were recorded in either study group after both treatments. In the coronectomy cases, the surgical site was primarily sutured. Primary closure improves post-operative bleeding management but may lead to more significant infection rates because a one-way valve allows food particles to reach into the socket but not easily get out [35].

Nevertheless, our patients did not experience any infectious complications, such as postoperative infection and alveolar osteitis. The rate of infectious complications in this study was low when compared to the incidence reported in previous studies of non-pathological MTMs, which have ranged from 2.8 to 30% after complete removal and from 0 to 14.6% after coronectomy [2, 3, 11, 31–33, 36]. Our study’s absence of alveolar osteitis is very positive, especially since smokers were included, who are more likely to acquire alveolar osteitis [36]. Our study methodology included taking great care to create bleeding and a coagulant in the socket. Patients were instructed not to rinse their mouth or spit during the first 24 h after surgery to avoid disrupting coagulant production. Additionally, the patient’s use of chlorhexidine mouthwash could have contributed to minimalizing the infectious complications. Chlorhexidine mouthwash has been proven to successfully lower the number of oral microbial populations (gram-positive and gram-negative bacteria and yeasts). It thus might be useful for reducing infectious complications [37–39].

The low complication rates in the dentigerous cyst group can be explained by a theory first described by Malden & D’Costa e Rego [40]. Theoretically, coronectomy is an appropriate approach for treating the cyst since it involves eliminating the crown below the dentin-enamel junction, where the dentigerous cyst originates [6, 8]. The cyst removal and its origin on the MTM during coronectomy prompt a wound-healing process similar to that observed following non-pathological MTM surgery.

Two patients (5%) who were diagnosed with a dentigerous cyst experienced temporary hypoesthesia of the IAN after coronectomy (*p* = 0.514). IANI is a postoperative complication, and previous studies have reported comparable rates (0–9.5%) after coronectomy of non-pathological MTMs [2, 3, 31, 41–47]. Both patients in our study recovered within six months, comparable with previous studies reviewing IANI after coronectomy of non-pathological MTMs [3, 33]. Notably, dentigerous cysts can potentially cause tooth displacement, resulting in compression or contusion of the IAN and hypo-anesthesia [8]. However, patients in our study did not experience numbness before surgery. Theoretically, the removal of the cyst could have resulted in postoperative hypoesthesia.

In this study, only two (2%) MTMs (both with caries) required re-surgery to remove retained roots completely. Previous studies of non-pathological MTMs show more significant re-surgery rates, varying from 0 to 11.3% [2, 3, 33, 42–47].

Our present results revealed that the administration of antibiotics was significantly correlated with the ASA score (Z = -2.390 and *p* = 0.017) and the extent of caries (Z = -2.502 and *p* = 0.012). These identified correlations align with the patient- and molar-related factors that the practitioners expected to be associated with risk for postoperative complications (large cysts, deep caries, and related health status). However, we found no significant relationship between antibiotic use and cyst size in either study group. This suggests a more flexible threshold for antibiotic prescription related to cyst size. Notably, since bacteria do not cause a dentigerous cyst, it is questionable whether antibiotics are required.

The prospective design of this study eliminates patient selection bias that could have affected the number of postoperative complications, such as the extent of the pathologies, impaction degree, and health status. Additionally, patient- and molar-related factors did not significantly influence our study’s number of postoperative complications.

The non-responder rate in the short-term results was minimal because the patients’ follow-up was based on self-reported problems, and all patients agreed to declare their postoperative complications. Subjects were observed at two-, six- and twelve months after surgery to assess complications and the need for secondary surgery after coronectomy. The most extended follow-up was four years and ten months.

This study also had limitations that must be discussed when interpreting the outcomes. As mentioned, the non-responder rate is considered minimal. However, since the data acquired from patients were mostly self-reported, patients who did not report their postoperative problems were not included in the analyses, which should be noted as a potential bias. Furthermore, seven patients did not attend their six-month examination after coronectomy, which contributed to attrition bias.

Another limitation applies to the selective prescription of postoperative antibiotics only for patients with large cysts, deep caries, and those with a related health status. Prescribing antibiotics for a selected group leads to discrepancies in both research groups and introduces a potential bias in the incidence of postoperative infections and persistent pain.

It is also essential to consider the IAN risk. Computer tomography scans were used to assess the likelihood of IANI and served as a guide for deciding whether to remove the MTM or perform a coronectomy. Since the roots of the mandibular third molars in the coronectomy group were closer to the IAN than those in the complete removal group, no reliable and fair comparison of the IANI incidence after both treatments can be made.

The most significant limitation of the study was the number of participants, which limited the number of complications after surgery. A larger patient group could provide more evidence regarding this subject.

In a previous study, only MTMs with caries limited to enamel or dentine were offered coronectomy to avoid avital dental pulpal tissue [13]. In contrast, our method included caries lesions extending into enamel, dentin, and pulp, which yielded postoperative complication rates of 0%, 3%, and 5%, respectively (not significantly different). Theoretically, a more extensive caries lesion may increase the risk of complications, considering that residual radices are not sterile. However, our study results do not confirm the belief that the surgical site must be sterile for a successful coronectomy [11]. A study with a larger sample size may provide more evidence to confirm our findings regarding the safety of coronectomy in MTMs with caries into the pulp.

## Conclusion

For pathologically impacted MTMs (with dentigerous cysts and caries), our present results support the short-term and long-term safety of coronectomy treatment. The rate of postoperative complications did not significantly differ between coronectomy and complete removal, suggesting that coronectomy can be indicated for pathological MTMs that are very close to the inferior alveolar nerve. For this practice to become standard care, further studies should be performed with extended patient inclusion- and a larger sample size.

## Data Availability

No datasets were generated or analysed during the current study.
